# 17-α Hydroxyprogesterone Nanoemulsifying Preconcentrate-Loaded Vaginal Tablet: A Novel Non-Invasive Approach for the Prevention of Preterm Birth

**DOI:** 10.3390/pharmaceutics11070335

**Published:** 2019-07-14

**Authors:** Manali Patki, Kiersten Giusto, Samir Gorasiya, Sandra E. Reznik, Ketan Patel

**Affiliations:** 1 Pharmaceutical Sciences, St. John’s University, Queens, NY 11439, USA; 2 Pathology and Obstetrics and Gynecology and Women’s Health, Montefiore Medical Center/Albert Einstein College of Medicine, Bronx, NY 10461, USA

**Keywords:** 17-α hydroxyprogesterone caproate, preterm birth, self-nanoemulsifying system, preterm labor, vaginal delivery, nanoparticle, vaginal tablet

## Abstract

Preterm birth (PTB) is a major cause of infant mortality in the United States and around the globe. Makena^®^—once-a-week intramuscular injection of 17-α Hydroxyprogesterone caproate (17P)—is the only FDA approved treatment for the prevention of PTB. Invasive delivery of 17P requires hospitalization and expert personnel for injection. Vaginal delivery of 17P would be preferable, because of high patient compliance, reduced systemic exposure, fewer side effects, and no need for hospitalization. The objective of the present study was to prepare and evaluate a self-nanoemulsifying vaginal tablet of 17P. A solid self-nanoemulsifying preconcentrate (S-SNEDDS) of 17P and dimethylacetamide (DMA) was developed using medium chain triglycerides, a non- immunogenic surfactant, and co-processed excipient (PVA-F100). The tablet prepared was characterized for emulsification time, particle size, solid state properties, and drug release. The formulation showed >50% inhibition of TNF-α release from LPS-stimulated RAW 264.7 cells. Importantly, there were significant differences in rates of PTB and average time to delivery between control and vaginal 17P-treated groups in LPS-stimulated timed pregnant E15.5 mice. Considering the lacuna of therapeutic approaches in this area, vaginal delivery of 17P for the prevention of preterm birth has significant clinical relevance.

## 1. Introduction

Prematurity is the leading cause of infant death and the second-leading cause of childhood death before the age of 5 [1]. A significant increase in preterm births (27%) over the last decade is a serious public health concern due to its dire consequences of increased infant mortality, morbidity, and socio-economic burden [1,2]. According to WHO, three-quarters of these deaths could be prevented with current, cost-effective interventions. Despite advances in healthcare, preterm birth and racial/ethnic disparities associated with it, remain a major challenge. African-American/non-Hispanic black infants disproportionately bear the burden of extreme prematurity (<28 weeks gestation). African-American women have 3.1 times the risk of preterm delivery and are almost five times more likely to deliver low birthweight infants [3,4]. In 2015, the rate of preterm birth among African-American women (13%) was about 50% higher than the rate of preterm birth among white women (9%). Research suggests that prenatal stress, including racism, is associated with an increased risk of poor birth outcomes—preterm birth and low birthweight. African-American women experience more chronic stress in their lives than white women, mostly due to racial discrimination [3,5]. Further, risks of preterm birth and infant mortality are higher for black infants born to mothers residing in the most segregated cities and neighborhoods, and there is a positive correlation between preterm delivery and lower household income and medicaid eligibility. Recent estimates indicate that preterm births account for over $20 billion in United States health care costs [6].

Makena^®^—an intramuscular injection of 17-alpha hydroxyprogesterone caproate (17P)—is the only United States Food and Drug Administration (FDA) approved drug available on the market for the prevention of preterm labor [7]. Invasive delivery necessitates clinics/expert healthcare personnel for injection, which eliminates the possibility of self-medication. Pain/soreness/itching at the site of injections is also a major complaint with 17P intramuscular (IM) injection, although somewhat less so with the novel approach of injecting subcutaneously [8]. Systemic toxicities and allergic reaction at the site of injection are additional concerns with the use of 17P injection [9,10]. Although the risk-to-benefit ratio for IM injection is low, a non-invasive delivery strategy would be a promising alternative for pregnant women with limited access to a clinical set-up or personnel, who live in distant neighborhoods or segregated areas (from the global perspective this is one of the biggest concerns), and who are not comfortable with IM injection. In 2003, Meis, et al. published results from a multicenter, prospective, double-blind, randomized controlled trial demonstrating that weekly treatment of 17P reduces recurrent PTB by approximately one-third [11]. 17P appears to be most efficacious in prolonging pregnancy in women with a previous early spontaneous PTB (<34 weeks gestation). At a weekly dose of 250 mg, 17P is associated with significantly lower incidences of recurrent spontaneous PTB and adverse neonatal outcomes compared with placebo. Weekly injections may be successfully administered in-office or in the woman’s home. 17P currently exists in various forms; a compounded 17P, branded Makena^®^, preservative free generic Makena^®^, and subcutaneous auto-injection [10,12]. Women living in distant neighborhoods or in areas with limited access to clinics in particular cannot take advantage of IM injections. Moreover, weekly IM injection is not a practical approach for the majority of high-risk women in developing countries in Asia, the Middle East, Africa, etc. [13]. Along with the limitations of the parenteral route of administration stated, limited shelf life of compounded or branded Makena^®^ further restrict the benefits of 17P among women with poor access to clinical settings worldwide. Makena^®^ is undoubtedly the only approved product for the prevention of preterm birth, but the drawbacks/limitations of Makena^®^ necessitate the availability of an alternative route or drug delivery platform. Owing to the lipophilicity of 17P, a lipid-based formulation, especially a self-nanoemulsifying drug delivery system (SNEDDS), would be advantageous by improving the solubility and bioavailability of the drug. SNEDDS offers the flexibility of converting a liquid into a solid dosage form by either filling a capsule with them or compressing them into a tablet [14,15]. Most importantly, excipients used to prepare SNEDDS are generally regarded as of safe (GRAS) status by the Food and Drug Administration (FDA).

Non-invasive, vaginal delivery of 17P is a preferable alternative because of its salient advantages including painless delivery, self-medication, high patient compliance, less systemic exposure (uterine first pass effect), and no need for clinics/expert personnel for injection [16,17,18]. Vaginal administration can address the problems associated with IM or subcutaneous injection of 17P with minimal systemic toxicity. From the perspective of cost, self-medication, intellectual property protection, large-scale manufacturing, storage conditions, and availability, a vaginal tablet would be a practical and viable approach. There is no previous report of either a preclinical or clinical investigation of vaginal delivery of a 17P-loaded nanoformulation. The main objective of this paper is to develop and characterize a 17P-loaded vaginal tablet and evaluate in vivo efficacy using a mouse model of preterm birth.

## 2. Materials and Methods

### 2.1. Materials

17P was procured from Santa Cruz Biotechnology Inc. (Dallas, TX, USA). Lipopolysaccharide from *Escherichia coli* O111:B4 was obtained from Sigma Aldrich (St. Louis, MO, USA). Dimethylacetamide (DMA) was purchased from Sigma Aldrich, USA. Medium chain triglyceride (MCT) Captex 300, Parteck MXP and Kolliphor HS 15 (PEG-15-Hydroxystrearate) were obtained as a gift sample from Abitec Corporation (Columbus, OH, USA), Millipore Sigma (Burlington, MA, USA), and BASF (Florham Park, NJ, USA), respectively. Florite 100 was received as a gift sample from Tomita Pharmaceutical Company Ltd. (Fort Lee NJ, USA). KG 1000 was received as a gift sample from Asahi Kasei corporation (Tokyo, Japan). High performance liquid chromatography (HPLC) grade water, acetonitrile, and methanol were obtained from Fisher Scientific (Hampton, NH, USA).

### 2.2. Analytical Method

A Waters Alliance system equipped with a 2998 PDA detector and Hypersil ODS column (250 mm × 4.6 mm, 5 µm) was used for chromatographic separation of 17P. The mobile phase was acetonitrile:water (80:20) at a flow rate of 1.2 mL/min [19]. For analysis, samples were loaded into the HPLC auto-sampler using the Empower 3 software and the output signal was monitored and processed. The column temperature was kept at 25 °C and retention time of 17P was found to be 7.3 ± 0.1 min detected at 242 nm. Linearity equation for HPLC was Y = 38451X + 15913 with R^2^ of 0.9998.

### 2.3. Preparation of Simulated Vaginal Fluid

Simulated vaginal fluid (SVF) was prepared to evaluate the solubility and drug release of 17P and 17P-loaded SNEDDS. SVF was prepared as described by Owen et al. (1999). To prepare 500 mL of SVF, glucose (2.5 g), bovine serum albumin (0.009 g), glycerol (0.08 g), sodium chloride (1.75 g), potassium hydroxide (0.7 g), calcium hydroxide (0.11 g), lactic acid (1 g), acetic acid (0.5 g), urea (0.2 g), and water were added in a beaker and stirred mechanically until a clear solution was formed. The pH of the final solution was adjusted to 4.5 using 0.1 N HCl.

### 2.4. Development of 17P-Loaded Self-Nanoemulsifying Preconcentrate (SNEDDS)

17P-loaded SNEDDS was prepared using dimethylacetamide (DMA), medium chain triglycerides (MCT), a non-immunogenic surfactant (Kolliphor HS 15). For the preparation of 17P-loaded SNEDDS, 17P was solubilized in DMA and added in the mixture of Kolliphor HS 15 and MCT at 50 °C. A clear solution was obtained with the help of a vortex mixer. The prepared SNEDDS was dispersed in water to assess its emulsification. To optimize the DMA concentration, the formulation was prepared using 0% DMA, 14% DMA, 40% DMA. The prepared formulations were added to 20 mL of SVF. At each time point, precipitated 17P was separated from nanoglobules using centrifugation (10,000 rpm, 10 min). A volume of 50 µL of clear supernatant was collected and mixed with the HPLC mobile phase for evaluating the dissolved concentration of 17P in SNEDDS. A graph of % 17P in solution versus time was plotted. The particle size and polydispersity index of different formulations prepared were determined using DLS particle size analyzer (Malvern Zetasizer Nano ZS, Malvern, UK). The preconcentrate was diluted with HPLC-grade water to ascertain that the light scattering intensity was within the optimum range of the instrument’s sensitivity. The diluted samples were placed into the cuvette and the data were analyzed. All experiments were carried out in triplicate.

### 2.5. Preparation of Solid 17P-Loaded SNEDDS (S-SNEDDS)

17P SNEDDS was adsorbed on co-processed excipient (CPE) prepared using polyvinyl alcohol (PVA) and mesoporous calcium silicate (Florite 100) as described previously [19]. Optimized SNEDDS was adsorbed onto the selected CPE in a 1:1 *w*/*w* ratio. SNEDDS was slowly added to the CPE in mortar pestle with continuous mixing to prevent any lump formation. A free-flowing powder was obtained at the end of the mixing process.

### 2.6. Solid State Characterization of S-SNEDDS

DSC studies were performed using a Q200 modulated DSC instrument (TA Instruments, New Castle, DE, USA). Six mg of samples were weighed in a pan and were sealed hermetically. Equilibration of the samples was carried out at 25 °C and the samples were heated from 30 to 200 °C at a scanning rate of 10 °C/min. The data was analyzed using TA Instruments universal analysis 2000 software. X-ray powder scattering measurements of drug, F100, PVA, and 17P S-SNEDDS were recorded using a Shimadzu XRD-6000 Diffractometer (Shimadzu, Kyoto, Japan). The diffractometer is equipped with a monochromatic Cu-Kα radiation source and nickel filter. PXRD diffraction patterns were recorded using the scanning range (2θ) from 0° to 60° at the rate of 2°/min.

### 2.7. Preparation and Characterization of a Vaginal Tablet

S-SNEDDS was converted into a vaginal tablet by the direct compression method. S-SNEDDS was mixed with directly compressible diluents like microcrystalline cellulose, Kollidon CL (superdisintegrant) and lubricant. The mixture was compressed at pressures ranging from 2500 to 7000 lbs using a 10 mm flat face punch (Natoli Engineering, Totowa, NJ, USA) on a single punch Carver Press assembly (Carver Inc, Wabash, IN, USA). As shown in Table 1, different combinations of S-SNEDDS and KG 1000 were mixed, keeping the disintegrant and magnesium stearate concentration constant (i.e., 5% *w*/*w* and 0.4%* w*/*w*, respectively).

### 2.8. Characterization of Vaginal Tablet

Tablets were characterized for hardness, thickness, friability, disintegration time, and drug release. A pharmaceutical hardness tester (Pharma Alliance Group, Valencia, CA, USA) was used for evaluating the hardness. The thickness of the tablet was determined using a digital vernier caliper. A friability test was performed using an HT-2 Friabilator USP (Sotax, Aesch, Switzerland). Tablets (*n* = 10) were weighed and placed in the friabilator and rotated at a speed of 25 rpm for 4 min as per United States Pharmacopeia (USP). The tablets were reweighed, and the percentage weight loss was calculated. The disintegration time was determined using a disintegration test apparatus, the USP Disintegration Apparatus (Pharma Alliance Group, Valencia, CA, USA) in 500 mL SVF. All measurements were done in triplicate.

### 2.9. In Vitro Drug Release

An in vitro release test of a 17P powder and a 17P SNEDDS-loaded tablet was performed, using a USP apparatus 2 (Distek Symphony 7100 bathless dissolution apparatus, Distek, North Brunswick Township, NJ, USA). The dissolution was carried out in 500 mL SVF with 0.5% *w*/*v* Tween 80 at 75 rpm and 37 ± 0.5 °C. A 2 mL sample was withdrawn periodically at 10, 20, 60, 90, 120, and 240 min. The samples were centrifuged at 12,000 rpm for 5 min, the supernatant was diluted with acetonitrile and the drug was immediately quantified using HPLC.

### 2.10. TNF-α Assay

The effect of the MCT and DMA on TNF-α release from lipopolysaccharide stimulated RAW cells was evaluated using a mouse TNF-α ELISA kit. RAW 264.7 cells were seeded at a density of 1.2 × 10^6^ cells per T25 cm^2^ tissue culture flask and incubated overnight at 37 °C and 5% CO_2_ in a complete growth media. The following day, various dilutions of SNEDDS were prepared in media and added to the flask at final concentrations of 0.5 mg/mL. After 2 h of incubation, cells were treated with 1 µg/mL LPS. At the end of a 24 h treatment, cell culture supernatants were collected, centrifuged at 1500× *g* for 5 min, and stored at −80 °C. Cell culture supernatants were analyzed to determine the levels of TNF-α using ELISA kits (Invitrogen, Fisher Scientific, Pittsburgh, PA, USA) as per the manufacturer’s protocol.

### 2.11. Animals

Eight-week-old female and male Swiss Webster mice were purchased from Taconic Farms (Rensselaer, NY, USA). Mice were housed in cages (two mice per cage) with a temperature of 23 ± 1 °C and humidity of 50 ± 10% on a 12:12 h light/dark cycle. Mice were given tap water and standard laboratory chow ad libitum. All experimental protocols were approved by the St. John’s University Institutional Animal Care and Use Committee (Identification# 1910, approval: 3 May 2017). In addition, research was conducted according to the National Institutes of Health Guide for the Care and Use of Laboratory Animals.

### 2.12. In Vivo Efficacy Testing in a Preterm Birth Mouse Model

LPS induced preterm birth in mice is a well-established and relevant model for such investigation [20,21,22,23]. Timed pregnant Swiss Webster mice (*n* = 11) were injected intraperitoneally on E15.5 (*t* = 0) with LPS (serotype 026:B6, obtained from Sigma, 25 mg/kg) dissolved in PBS to induce preterm labor. Mice were randomly assigned to 2 groups and given vaginal administrations of 30 μL Blank formulation (*n* = 6) or 17P SNEDDS (*n* = 5), using a 200 μL micropipette, at *t* = −8, 1, and 8 h. Blank formulation contains all the excipients in the same ratio as optimized formulation but no 17P. Mice were continuously monitored for time of delivery and the number of pups dropped was recorded. In accordance with the St. John’s University Institutional Animal Care and Use Committee, mice were sacrificed at *t* = 24 h. The mice were necropsied at the end of experiments to confirm pregnancy and to determine the number of pups retained in utero.

### 2.13. Statistics

The data are shown as the mean ± standard deviation (SD). Student’s *t*-test or one-way ANOVA was used to determine the significance of the differences among treatment groups using GraphPad Prism version 5.0 (San Jose, CA, USA) and a value of *p* < 0.05 was considered statistically significant.

## 3. Results

### 3.1. Development of Self-Nanoemulsifying Preconcentrate

**Figure 1:**
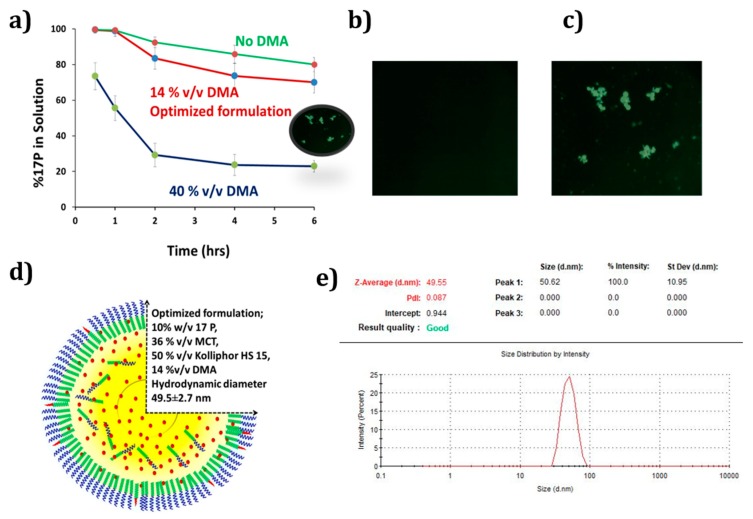
Characterization and optimization of 17P self nano emulsifying drug delivering system (SNEDDS). (**a**) Effect of dimethylacetamide (DMA) on physical stability of 17P-loaded SNEDDS. The addition of 14% DMA helped in maintaining the concentration of 17P up to 6 h. Increasing DMA concentration to 40% led to a rapid precipitation of 17P from solution; (**b**) microscopic image of 17P SNEDDS with 14% DMA (4× magnification); (**c**) microscopic image of 17P SNEDDS with 40% DMA. The addition of 14% DMA showed no precipitation, whereas increasing the DMA concentration to 40% led to the precipitation of 17P (4× magnification); (**d**) optimized SNEDDS composition; (**e**) Particle size analysis of 17P.

Considering the physicochemical properties of 17P, SNEDDS was prepared using medium chain triglycerides, the non-immunogenic surfactant Kolliphor HS 15 and a co-solvent—DMA. The overall drug loading of DMA-free batches was two times lower than the batches with 10% DMA. Moreover, DMA facilitated spontaneous emulsification and substantially lowered the emulsification time. Batches with 0% DMA took 5–7 min to emulsify while batches with 10% DMA emulsified in 2–3 min. On the other hand, due to the polar nature and co-solvency effect of DMA, batches containing DMA showed higher precipitation of 17P compared to DMA-free batches. As shown in Figure 1a, as DMA percentage increases from 14% to 40% *v*/*v*, the amount of 17P in solution decreases from around 82.5% to 25.2% in 2 h. Whereas when no DMA was used, the amount of 17P in the solution was 90% at 2 h. As shown in Figure 1b,c, microscopic images taken within 2 h confirmed that solution with 14% DMA showed no precipitation, whereas increasing the DMA concentration to 40% led to the precipitation of 17P. Thus, based on the rate of precipitation, emulsification time and particle size, the SNEDDS was optimized. The optimized SNEDDS has 10% *w*/*v* 17P, 14% *v*/*v* DMA, 36% *v*/*v* MCT, 50% *v*/*v* Kolliphor HS 15 as shown in Figure 1d. Figure 1e represents the particle size of the optimized SNEDDS of 49.55 ± 2.7 nm and a monomodal peak with a polydispersity index of 0.087. The zeta potential was found to be −6.93 mV. Optimized SNEDDS with 10% *w*/*v* 17P loading was adsorbed onto the co-processed excipient PVA-F100 at a 1:1 *w*/*w* ratio. A free-flowing powder was obtained at the end of the mixing process.

### 3.2. Solid State Characterization

**Figure 2:**
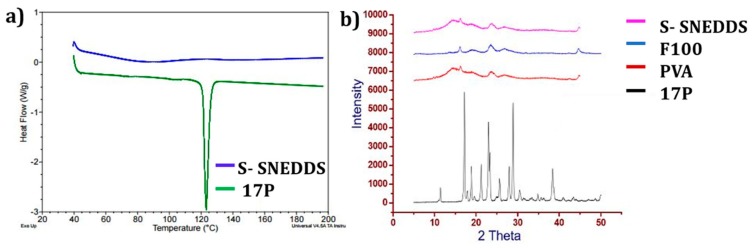
Solid state characterization of 17P and S-SNEDDS. (**a**) Differential scanning calorimetry (DSC) thermogram of 17P and S-SNEDD; (**b**) XRD of polyvinyl alcohol (PVA), F-100, 17P, S-SNEDDS. An absence of a sharp endothermic peak in DSC and a characteristic peak in XRD in S-SNEDDS confirmed that 17P remained in a solubilized state after adsorption of SNEDDS on PVA-F100.

DSC endotherms of 17P and 17P formulation (S-SNEDDS) are shown in Figure 2a. 17P showed a sharp melting endotherm at 121 °C, representing the melting point of crystalline 17P. An absence of sharp endothermic peak in DSC in adsorbed samples confirmed that 17P remained in a solubilized state after adsorption of SNEDDS on CPE. Additionally, the absence of a crystalline peak in XRD in Figure 2b confirmed that 17P remained in a solubilized state after adsorption of SNEDDS on PVA-F100.

### 3.3. Preparation and Characterization of Vaginal Tablet

Based on the hardness, friability, and disintegration time, the batch was optimized. Our target value for hardness and friability was 4–6 kg and <1%, respectively. As shown in Table 1, initially tablets were prepared using a pressure up to 2500 lbs. It was found that, irrespective of the ratio of adsorbed S-SNEDDS:KG 1000, the tablets were friable. Later the powder was compressed at a higher pressure of 6000 lbs. Batches F2 and F5 gave a hardness of 1.6 and 4.7 kg, respectively. However, with a further increase in the pressure (7000 lbs), tablet hardness increased up to 8.1 kg. Despite F6 showing hardness of 8.1 kg, it was not selected since the disintegration time of the F6 batch was >45 min. Additionally, there was oozing out of the liquid preconcentrate after compression at a higher pressure, which was visible on the surface of the tablet.

The composition of the optimized vaginal tablet is presented in Table 2. The optimized tablet (F5) hardness was found to be 4.7 ± 1.2 kg with a thickness of 3.9 ± 0.1 mm. Friability was found to be less than 1% and it quickly disintegrated within 5 min.

### 3.4. In Vitro Drug Release

**Figure 3:**
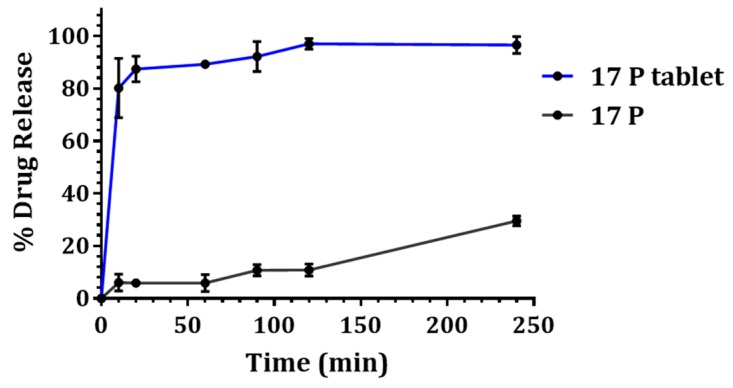
In vitro drug release of 17P tablet and API in 500 mL simulated vaginal fluid (SVF) + 0.5% *w*/*v* Tween 80. Within 10 min 17P release from the tablet was found to be around 80%, whereas the release from API was only 5%. 17P tablet helped maintain the drug in a supersaturated state up to 4 h.

As shown in Figure 3, 17P showed faster dissolution from the tablet compared to the API alone. Within 10 min, the percentage of 17P dissolved from the tablet was found to be around 16 times higher compared to API. Within 2 h, the vaginal tablet showed nearly complete release of 97%, whereas the API showed mere 10% drug release. Further, in the case of the tablet, even after 4 h the release of 17P was found to remain constant, confirming the complete solubilization of 17P along with negligible precipitation in the dissolution media.

### 3.5. TNF-α Study

**Figure 4:**
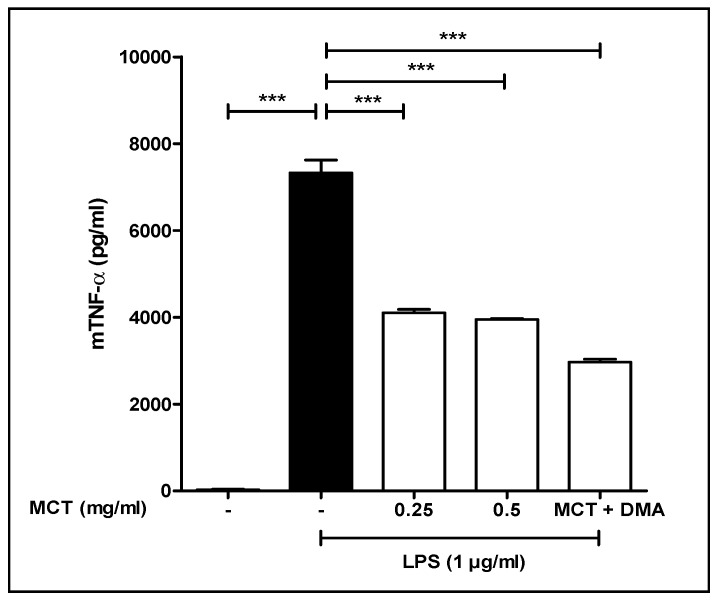
Effect of medium chain triglyceride (MCT) and MCT + DMA on LPS-stimulated TNF-α secretion from RAW 264.7 cells. MCT at 0.25 and 0.5 mg/mL reduced TNF-α secretion by 46% and 50%, respectively. The combination of MCT + DMA reduced TNF-α secretion by 60%. (*** *p* < 0.001, ANOVA).

As shown in Figure 4, LPS was added to the cells to induce the release of TNF-α. MCT concentrations of 0.25 and 0.5 µg/mL decreased TNF-α release by 46% and 50%, respectively. When DMA was added along with the MCT, the decrease in TNF-α was found to be 60%, showing that DMA further decreased the release of TNF-α. Our results are in agreement with previously reported results showing that DMA inhibits TNF-α release [24,25]. The reduction of cytokine release produced by the MCT and DMA may further enhance the formulation’s efficacy in preventing PTB.

### 3.6. Vaginal Administration of a Hydroxyprogesterone Nanoformulation Delays the Onset of Preterm Labor (PTL) in LPS-Stimulated Pregnant Mice

**Figure 5:**
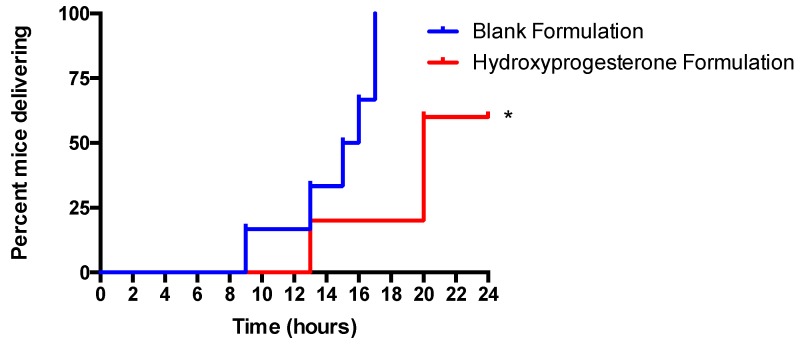
17P SNEDDS reduces the incidence of preterm birth and delays labor and delivery in LPS-stimulated timed pregnant E 15.5 mice. Mice injected with 25 mg/kg of LPS to induce PTB were treated with vaginal administration of the17P formulation. All mice were observed for PTB for a period of 24 h. 17P significantly reduced the percentage of mice delivering over time. * *p* < 0.05 Mantel–Cox Test.

**Figure 6:**
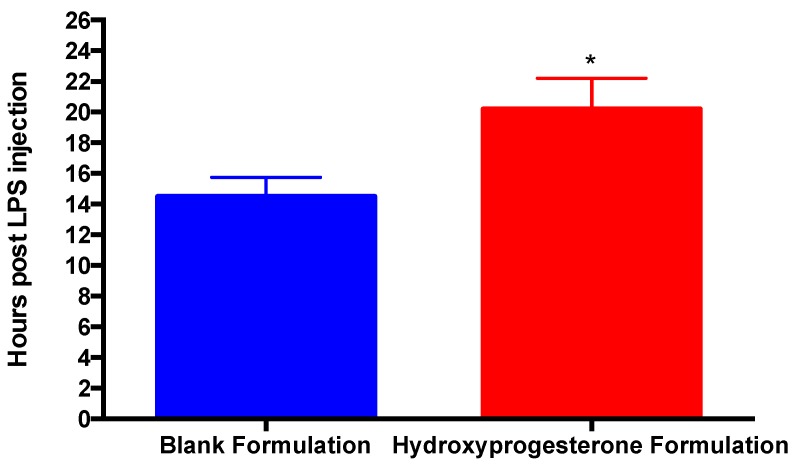
17P SNEDDS significantly increases the time to delivery compared to the Blank formulation group. Treatment with the 17P formulation significantly increased the hours post LPS injection to delivery. For the purpose of statistical analysis, a delivery time of 24 h was assumed for mice that did not deliver during the experiment. * *p* < 0.05, Student’s *t* test.

In the blank formulation control, six out of six mice delivered (100%) by the end of the 24 h experimental period (Figure 5). In the 17P formulation treatment group only three out of five mice (60%) delivered by the end of the 24 h experimental period, which was significantly lower than the control group (*p* < 0.05) (Figure 5). The average time to delivery for the blank formulation group was 14.5 h, while the average time to delivery for the 17P formulation group was 20.2 h (*p* < 0.05) (Figure 6). For the purpose of statistical analysis, a delivery time of 24 h was assigned to mice that did not deliver since the experiment had to be stopped at this time point. The gestation period of Swiss Webster mice is 19.5 days, while the gestation period of humans is 38 weeks or 266 days. The difference between the mean time of delivery of the negative control group and the mean time of delivery of the treated group is 5.7 h; therefore, equates to a delay in labor of approximately three days of a human gestation. However, this extremely conservative estimate assumes that the two mice that had not delivered by the end of the experiment at *t* = 24 h would have delivered immediately, had the experiment proceeded. In reality, those two mice may have delivered any time between *t* = 24 h and *t *= 96 h (the end of the normal gestation period). Thus, the mean time of the delivery of the treated group is any time between *t* = 20.2 h and *t* = 49 h and the delay in mean time of delivery in the treated group is any time between 5.7 and 34.5 h. A period of 34.5 h of mouse gestation equates to approximately 19.6 days of human gestation. The LPS was administered at E15.5, which equates to approximately 30 weeks’ gestational age in humans. Therefore, translating our results from mice to humans, the mean time of delivery was shifted from approximately 31 weeks and 1 day’s gestational age to approximately 31 weeks and 4 days, at a minimum, and up to 33 weeks and 6 days, at a maximum.

## 4. Discussion

An intramuscular injection of 17-alpha hydroxyprogesterone caproate (17P) is the only therapeutic intervention for prevention of preterm birth in women with a history of a previous spontaneous singleton preterm birth. For the population with limited or no access to the 17P oil-based injection, and for the women who do no prefer IM injections, an alternative drug delivery platform is a dire necessity. In this paper, we have developed a vaginal tablet loaded with a self-nanoemulsifying preconcentrate of 17P. After characterizing our SNEDDS formulation for various physicochemical parameters, we converted the liquid preparation into a solid unit dosage form—a tablet. Further, the preconcentrate was evaluated in an in vivo model of preterm birth and the formulation was shown to reduce preterm birth by 40%.

Since 17P is a highly lipophilic molecule, a lipid-based formulation is ideal. However, a simple solution of 17P in oil, while suitable for sustained-release IM injection, is not suitable for vaginal delivery. A solution of 17P in oil will leak out of the vagina without absorption of the drug due to the extreme lipophilicity of both 17P and oil. Therefore, to enhance dissolution and permeability, an advanced formulation of 17P was prepared. The co-solvent and surfactant selected facilitated the formation of nano-sized globules of oil loaded with drug which come in contact with vaginal fluid. Nanoemulsion results in rapid dissolution of drugs in a physiological milieu and promotes permeation across membranes. Importantly, nanoglobules maintain supersaturation for four hours, which is essential for drug absorption. Hydrophobic drugs solubilized by co-solvents rapidly precipitate upon contact with aqueous media. Such precipitation limits the bioavailability of drugs. Here, nanoglobules served as a reservoir for 17P, promoting rapid exchange of drug molecules between oil drop, interface, and aqueous phase. At the same time, they prevented aggregation and precipitation of the drug. Thus, enhanced solubility and supersaturation created a high concentration gradient of the drug across the vaginal mucosa and led to rapid and high drug absorption [26,27,28,29]. Along with the nanosize of the globule, the ζ-potential was found to be negative, which is in agreement with the previous reports that state that a negative zeta potential is necessary for mucus penetration [30]. We hypothesize that vaginally-administered 17P will achieve high uterine concentrations due to the “uterine first pass effect” [31]. There are many reports demonstrating that lipophilic compounds are directly transported from the vagina to the uterus, which is the site of action for the prevention of preterm birth [32,33]. Moreover, due to its very high lipophilicity, 17P is expected to partition poorly into the systemic circulation. It is also known that lipophilic compounds have higher tissue residence times and; therefore, lower systemic exposure. Thus, targeted vaginal delivery will minimize the systemic side effects and toxicities of 17P.

Another advantage of preparing a vaginal tablet with advanced technology at a commercial scale is the minimization of compounding related errors. The currently available formulation—Makena injection (IM)—is a relatively simple formulation. Therefore, compounding pharmacies can easily prepare and dispense sterile compounded 17P, which they have done both before and after the introduction of Makena^®^ in 2011. However, there are many reports of the poor quality of compounded 17P, and there are mixed reviews on its potency and efficacy and on the purity of the active pharmaceutical ingredient [7,34]. Sterile compounding involves a great deal of risk compared to product manufacturing by a pharmaceutical company. In spite of the enforcement of USP <797> by the FDA, there are plenty of cases reported where poor compounding practice or compounding error resulted in severe consequences or deaths [7,35]. Very recently AMAG pharmaceuticals has introduced a single-dose generic version of preservative-free Makena^®^ to circumvent such issues. Recently, AMAG Pharmaceuticals received FDA approval for Makena^®^—Subcutaneous Auto-Injector. This is really a value added product, especially for women not comfortable with IM injections. However, issues of self-medication, shelf-life, accessibility, and cost associated with injection by professional health care personnel remain the same. A hypothetical pharmacoeconomic comparison revealed that the total cost of a 17P regimen would be reduced by at least 10-fold with self-medication compared to administration by professional healthcare personnel [36]. 17P SNEDDS-loaded vaginal tablets would be much easier to use and more affordable compared to both IM and SC injections. The vaginal route leads to a higher endometrial concentration compared to IM injection [37,38,39]. Moreover, due to the complexity of the technology used here, production in a compounding setting is not feasible.

There are several disadvantages of a liquid formulation, which include manufacturing, stability, transport, handling, and administration [40,41]. Filling a soft gelatin capsule with SNEDDS is the simplest approach, but the stringent storage conditions of capsules severely restrict their distribution into tropical or humid regions [42,43]. From the commercial and distribution standpoint, converting a SNEDDS into a free-flowable powder and then into a tablet is a much more viable option. SNEDDS are usually adsorbed onto inorganic silica to achieve a free-flowable powder [44,45]. However, incomplete release of drug from silica is the biggest limitation of such an approach. To circumvent this issue, we have designed a co-processed excipient which allows rapid emulsification of SNEDDS and complete dissolution. The development and optimization of co-processed excipient made of Florite^®^ 100 and PVA is described in our previous paper [46]. 17P SNEDDS-adsorbed co-processed silica was further mixed with a suitable diluent, disintegrant, and lubricant to prepare tablets. We prepared 10 mm tablets because commercial vaginal tablets have similar dimensions and tablets of 10 mm can be easily inserted into the vagina. Tween 80 was added into the dissolution medium (SVF) to maintain sink condition. There are various reports demonstrating the use of 250 to 900 mL of dissolution medium with surfactant to maintain sink condition for the characterization of vaginal tablets [47,48].

**Figure 7:**
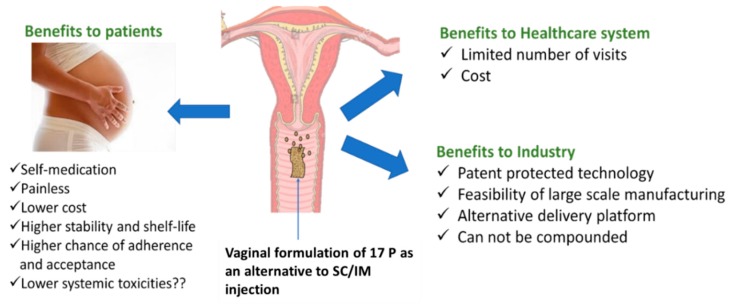
Schematic describing advantages of vaginal delivery of 17P.

According to the World Health Organization (WHO), more than 60% of preterm births occur in Africa and South Asia. A vaginal product would be very helpful for women of those countries where parenteral injection of 17P is not available or not feasible. As far as stability in global weather conditions and long-term storage, a vaginal tablet formulation would be a more viable option. The benefits of vaginal formulation over SC/IM injection are described in Figure 7.

## 5. Conclusions

A SNEDDS of 17P was developed using dimethylacetamide, medium-chain triglycerides and a non-immunogenic surfactant kolliphor HS15. Conversion of the liquid SNEDDS into a vaginal tablet did not alter the emulsification or release of the drug. Solid-state characterization confirmed that 17P remained in a solubilized state after adsorption on PVA-F100. An in vivo efficacy study showed that vaginal delivery of 17P can prevent PTB. Vaginal delivery is a non-invasive and painless alternative/option to SC/IM injection. It would be helpful for women with poor access to clinical settings (living in distant neighborhoods or segregated areas) or not comfortable with IM injection. Most importantly, systemic side effects/toxicities associated with parentally-administered 17P would be minimized by vaginal administration. Thus, considering adherence, acceptability, efficacy, and affordability, the dearth of effective pharmacotherapy to prevent PTB and the devastating complications of PTB, a vaginal tablet that can prevent PTB may have a significant positive impact on human health.

## Figures and Tables

**Table 1:** Effect of pressure and amount of diluent on the hardness of vaginal tablet.

Batch	Pressure (lbs)	S-SNEDDS (mg)	KG 1000 (mg)	Hardness (kg)
F1	2500	150	170	-
F2	6000	150	170	1.6
F3	7000	150	170	3.1
F4	2500	150	200	-
F5	6000	150	200	4.7
F6	7000	150	200	8.1

**Table 2:** Composition of the optimized tablet batch.

Composition	Weight/Tablet
S-SNEDDS	150 mg
Microcrystalline cellulose (KG-1000)	200 mg
Kollidon CL	18.5 mg
Magnesium stearate	1.5 mg
